# Visceral Artery Aneurysms in Liver Transplant Candidates and in Patients after Liver Transplantation

**DOI:** 10.1371/journal.pone.0029544

**Published:** 2011-12-21

**Authors:** Umberto Maggi, Daniele Dondossola, Dario Consonni, Stefano Gatti, Rossella Arnoldi, Manuela Bossi, Giorgio Rossi

**Affiliations:** 1 U.O. Chirurgia Generale e Trapianti di Fegato - Fondazione IRCCS Ca' Granda Ospedale Maggiore Policlinico, Milano, Italy; 2 U.O. Epidemiologia - Fondazione IRCCS Ca' Granda Ospedale Maggiore Policlinico, Milano, Italy; University of Colorado, United States of America

## Abstract

There are only few reviews concerning visceral aneurysms in cirrhotics, and a small number of papers on visceral aneurysms in liver transplant patients. The present paper investigates this condition in both groups of patients in a 10-year-retrospective study.

## Introduction

Visceral aneurysms are rare in the general population (rate<0.2%) [1]. Their detection lies between 7% and 50% of patients with liver cirrhosis [2]–[3]. The average age of occurrence varies from 49 [4] to 66 years [5]. Visceral aneurysms and pseudoaneurysms after liver transplantation (LT) represent a rare disease but the overall number of studied liver transplanted patients so far is very small. A review of the literature enabled us to identify approximately 60 post-transplant pseudoaneurysms of the hepatic artery (HA) and 7 splenic artery (SA) aneurysms [6–7–8–9–10], with an incidence estimated to be about 0.3–1% [6].

The aim of this study is to examine separately the visceral aneurysms and pseudoaneurysms treated in our unit over the past 10 years in two different groups of patients: patients with end-stage liver diseases – considered as representatives of patients with cirrhosis, even though at an advanced stage - and patients who underwent a LT.

Starting with a review of the published literature on this subject, we tried to identify the actual prevalence of visceral aneurysms and pseudoaneurysms, the possible causes of their development in these two groups of patients, and their correct treatment.

## Methods

### Step One: pre-transplant patients

As inclusion criteria we considered all consecutive adult patients with cirrhosis who underwent LT in our unit from January 2000 to November 2010 (n = 302); in these patients we calculated the pre-LT incidence of visceral aneurysms.

At the end, we included in this study only patients with hepatocellular cirrhosis with or without cancer and cholestatic cirrhosis. Patients with fulminant liver failure or other indications for LT, were excluded.

In these patients we attempted to identify potential common factors related to the occurrence of such aneurysms. For this part of the study, 3 further patients who underwent LT in 1995, 1996 and 1999 were considered. These 3 patients were not considered for the incidence of aneurysms, but they were for the study of the risk factors. Therefore 305 cases were studied. Among continuous variables there were: patient age, biochemical data (recorded at the time of transplantation) including serum Aspartate aminotransferase (AST), Alanine aminotransferase (ALT), Total Bilirubin, Creatinine, gamma -Glutamyltransferase (GGT), amylase, International Normalized Ratio (INR), blood platelets, AST platelet ratio index (A.P.R.I.) and Mayo End-Stage Liver Disease (MELD) Scores, and the longitudinal diameter of the spleen measured with the US scan.

Among categorical data, in addition to the epidemiological variables such as age and sex, we considered factors related to arterial and portal hypertension: we therefore evaluated the recipient gender, the presence in the medical history of previous surgery or a TACE (transarterial chemo-embolization), arterial hypertension, antihypertensive therapy, beta-blocker therapy, oesophageal varices, the type of liver disease, and the type of viral liver disease (hepatitis B and C).

### Step Two: post-transplant patients

We considered the visceral aneurysms and pseudoaneurysms in adult patients after the first LT. The LT itself was considered the main inclusion criteria for this part of the study, indipendently of the primary patology. Retransplantations and patients with known pre-LT aneurysms were excluded from the analysis. Therefore, 331 patients, transplanted from January 2000 to November 2010, were included in the study. The mean follow-up period was 3,9±3,1 years. The patients underwent abdominal US scan on the 1^st^ postoperative day and after that twice a week for the first month and then during the outpatient clinic appointments whose frequency decreased over time. However the US scan was performed whenever needed according to the clinical picture. The control CT scan was performed one month after surgery and after that whenever needed. The late-follow-up appointments were scheduled twice a year. No patients were lost during the follow up. Variables of liver donors, recipients and transplantations, possibly related to the onset of aneurysms and pseudoaneurysms, were identified. In this analysis we included also two cases of aneurysms that occurred in the post-transplant period just prior to the stated analysis (LT 315 and 444).

The variables analyzed in the 333 patients were as follows: continuous data including the donor's age, GGT, the recipient's age, biochemical data (recorded at the time of transplantation) – AST, ALT, total bilirubin, creatinine, GGT, amylase, platelets, INR –, the MELD score, the pre-LT longitudinal diameter of spleen detected on US scan, and the total ischemia time during transplantation.

Among categorical data, we considered the donor's and recipient's gender, the presence in the medical history of a previous surgery, TACE (transarterial chemo-embolization), pre-LT arterial hypertension, antihypertensive therapy, beta-blocker therapy, and oesophageal varices, the onset of pre-LT aneurysms, the type of liver disease and of viral liver disease (hepatitis B and/or C), the type of liver grafts (whole/partial), the performance of complex vascular reconstructions during the transplantation.

The study was approved by the Ethics Committee of the Institution where data were collected.

Informed consent was obtained as usual for medical, surgical, radiological treatments, not specifically for this retrospective study. Patients gave written consent for every procedure performed in the hospital including treatment of data for medical purposes.

### Statistical Analysis

In both steps we performed univariate analyses with the Student's t-test or χ2 test, whenever appropriate. We calculated the odds ratio (OR) and 95% confidence intervals (95% CI) of aneurysm using multivariate logistic models including variables that were significant on univariate analyses or were considered important in literature.

P≤0,1 was considered significant in the univariate analyses and ≤0.05 in the multivariate.

The Statistical analysis was performed using Stata 11 (StataCorp, 2009).

## Results

### Pre-transplant patients

Eight (2.6%) out of 302 end-stage chronic liver disease patients who underwent LT between 2000 and 2010 were identified as affected with visceral aneurysms before LT. Among the 3 patients added in the clinical analysis there was one patient with a pre-LT aneurysm (LT 272). Therefore, a total of 9 aneurysms were included in the clinical analysis, outside the calculation of incidence. Among cirrhotic, cholestatic and cancer (in cirrhosis) patients we observed 4 (2,3%), 1 (3,7%) and 3 (2,8%) aneurysms respectively.

There were 172 patients (56,4%) with cirrhosis caused by different pathologies, 27 patients (8,9%) with cholestatic cirrhosis and 106 patients (34,8%) with hepatocarcinomas in cirrhosis.

Males were 212 (69,5%), females 93 (30,5%).

In all 9 cases, the aneurysms were located in the distal segment of the SA, and in one case also in the middle one. In 4 patients aneurysms were multiple, in 5 single. The diameter of the aneurysms was between 1.5 and 3 cm.

At univariate analysis of continuous data, longitudinal diameter of spleen (p = 0.06) and hyperamylasemia (p = 0.017) were significantly higher in patients with aneurysms (Table 1). Categorical data showed statistical significance in the female group (odds ratio 2.9, p = 0.09), in the β-blocker therapy group (odds ratio 4.6, p = 0.015) and in the pre-LT TACE group (odds ratio 6.5, p = 0.017) (Table 2).

**Table 1:** Univariate analysis of continuous variables among cirrhotics waiting for LT, with and without visceral aneurysms.

continuous variables	Cirrhotics with visceral aneurysms		Cirrhotics without visceral aneurysms		
	N	mean±SD	n	Mean±SD	p
Age (years)	9	50±10	296	51±9	0,83
Total Bilirubin (mg/dl)	9	2,3±1,5	288	5±9,5	0,38
Creatinine (mf/dl)	9	0,7±0,3	269	1±0,7	0,30
INR	9	1,2±0,2	283	1,3±0,4	0,62
MELD Score	9	12±3	267	13±5	0,42
PLT (n/mm3)	8	56.000±22.017	253	82.620±51.226	0,12
Bipolar diameter of the spleen(cm.)	7	19,7±1,2	280	16,8±4	0,06
ALT (UI/L)	9	53±52	252	79±112	0,49
AST (UI/L)	9	69±54	252	98±144	0,55
GGT (UI/L)	9	55±42	237	88±87	0,26
Amylases (IU/L)	8	127±88	185	64±71	0,017
A.P.R.I.	9	3,8±3,9	252	4,9±12	0,81

**Table 2:** Univariate analysis of categorical variables among cirrhotics waiting for LT with and without visceral aneurysms.

			Cirrhotics with visceral aneurysms		Cirrhotics without visceral aneurysms		
Cat.Variables	categories	N	n	%	n	%	P
Rec. Gender	M	305	4	44,4	208	70,3	0,09
	F		5	55,6	88	29,7	
surgery pre-LT	yes	292	0	0	16	5,7	0,46
	no		9	100	267	94,3	
TACE	yes	287	2	33,3	20	7,1	0,01
	no		4	66,7	261	92,9	
HTA	yes	278	1	11,1	29	10,8	0,97
	no		8	88,9	240	89,2	
anti-HTA Treatment	yes	278	1	11,1	22	8,2	0,75
	no		8	88,9	247	91,8	
β-block. therapy	yes	292	5	55,6	60	21,2	0,01
	no		4	44,4	223	78,8	
Oesophageal varices	no	283	3	75	99	35,5	0,25
	F1		1	25	151	54,1	
	F2		0	0	0	0	
	F3		0	0	29	10,4	
Hepatic diseases	cirrhosis	305	4	44,4	168	56,8	0,76
	cholestatic		1	11,1	26	8,8	
	neoplastic		4	44,4	102	34,5	
HCV	yes	305	5	55,6	146	49,3	0,71
	no		4	44,5	150	50,7	
HBV	yes	305	4	44,5	89	30,1	0,35
	no		5	55,6	207	69,9	
Viral diseases (HCV/HBV)	yes	305	8	88,9	222	75	0,34
	no		1	11,1	74	25	

The multivariate analysis (Table 3) showed elevated ORs in the β-blocker therapy group, increased amylase group and pre-LT TACE group.

**Table 3:** Risk of pre-LT aneurisms among cirrhotics waiting fort LT transplant.

	ODDS RATIO	95% CI	P
TACE	11,76	1,46 – 94,4	0,02
β-block. therapy	7,49	0,98 – 57,7	0,05
Amylases (x 100)	1,95	1,07 – 3,56	0,02

Results of a multivariate logistic regression.

Splenic aneurysms in cirrhotic patients were always asymptomatic. No spontaneous pre-LT aneurysm rupture was observed. The diagnosis of aneurysm in these patients was always made with CT scan during the pre-LT study.

Four patients (44%) underwent a proximal SA ligation during LT (in 1 case the proximal stump of the SA was used for the anastomosis with the common HA of the donor).

Five patients (55%) underwent splenectomy as treatment for splenic aneurysm, and in particular three during LT.

In a fourth patient no treatment could be performed during LT and a postoperative embolization of the SA failed; a splenectomy was performed 2 months later. In a fifth patient an urgent post LT splenectomy was required for ruptured aneurysm.

### Post-transplant patients

We examined 331 patients who underwent consecutive LTs from January 2000 to November 2010: 223 were males (67.4%) and 108 (32.6%) females, the average age was 50.5±10 years; the follow-up period was 3.9±3.1 years.

Causes of LT were: mixed cirrhosis in 170 (51.3%), cholestatic cirrhosis in 26 (7.8%), hepatocarcinoma in cirrhosis in 109 (32.9%), fulminant hepatic failure in 15 (4.5%), and other disorders in 11 (3.3%).

Visceral aneurysms and pseudoaneurysms were detected in 6 patients (1.8%). Two additional patients with post-LT aneurysms identified just before the study period (one case in 1996, the other in 1999) were included only for the statistical analysis of aetiopathogenetic variables. Consequently, the total number of patients affected by aneurysms and pseudoaneurysms in the postoperative period was 8: there were 5 SA aneurysms and 3 pseudoaneurysms of the HA.

Aneurysms occurred in 4 patients (2,3%) with mixed cirrhosis, 1 patient (3,8%) with cholestatic cirrhosis, 2 patients (1,8%) with hepatocarcinoma, and 1 patient (9%) with other disorders (a case of Budd-Chiari Syndrome).

In univariate analysis (Table 4–5), only the female group reached statistical significance (OR 3.5 – 95% CI 0,83–15,1, p = 0.06).

**Table 4:** Univariate analysis of continuous variables (just before LT) of transplanted patients, according to the presence of post-LT visceral aneurysms.

CONTINUOUS VARIABLES (t-test) at LT					
	PATIENTS W POST_LT ANEURYSMS		PATIENTS W/O ANEURYSMS		
	n	Mean±SD	n	Mean±SD	p
D. Age (years)	8	46±22	324	50±18	0,58
D. GGT (IU/L)	8	49±74	282	47±70	0,93
Total ischemia time (min.)	8	515±115	324	512±107	0,94
R. Age (years)	8	45±14	325	50±10	0,19
R. Total Bil.irubin (mg/dL)	8	5,2±5,8	315	5,5±10,2	0,93
R. Creatinine (mg/dL)	7	0,7±0,3	295	1,1±0,9	0,31
R. INR	8	1,4±0,2	310	1,4±0,6	0,90
R. MELD	6	12±3	294	14±6	0,46
R. PLT (n/mm3)	7	89.285±93.679	278	86.126±54.301	0,88
R. spleen bipolar diameter(cm.)	5	17±3	305	16±4	0,61
R. ALT (IU/L)	7	78±51	277	133±347	0,67
R. AST (IU/L)	7	82±41	277	134±335	0,68
R. GGT (IU/L)	7	58±46	258	88±86	0,35
R. Amylases (IU/L)	6	84±66	203	66±71	0,55

**Table 5:** Univariate analysis of categorical variables (just before LT) of transplanted patients, according to the presence of post-LT visceral aneurysms.

CATEGORICAL VARIABLES							
			PATIENTS W POST_LT ANEURYSMS		PATIENTS W/O POST_LT ANEURYSMS		
	categories	N	n	%	n	%	p
D Gender	M	322	5	62,5	157	50	0,48
	F		3	37,5	157	50	
Rec Gender	M	333	3	37,5	221	68	0,06
	F		5	62,5	104	32	
pre-LT Surgery	Y	317	1	12,5	16	5,2	0,36
	N		7	87,5	293	94,8	
pre-LT TACE	Y	313	1	16,7	22	7,2	0,37
	N		5	83,3	285	92,8	
pre-LT HTA	Y	303	1	12,5	33	11,2	0,90
	N		7	87,5	262	88,8	
pre-LT anti-HTA treatment	Y	303	1	12,5	26	8,8	0,71
	N		7	87,5	269	91,2	
pre-LT anti-β-blocK. treatment	Y		1	12,5	65	21	0,55
	N		7	87,5	244	79	
oesophageal varices	N	309	3	100	107	35	0,06
	F1		0	0	167	54,6	
	F2		0	0	0	0	
	F3		0	0	32	10,5	
Pre-LT Aneurysms	Y	333	1	12,5	7	2,2	0,05
	N		7	87,5	318	97,8	
Hepatic diseases	cirrhosis	333	4	50	167	51,4	0,58
	colestatic		1	12,5	26	8	
	neoplastic		2	25	107	32,9	
	fulminant		0	0	15	4,6	
	others		1	12,5	10	3,1	
HCV	Y	333	3	37,5	148	45,5	0,65
	N		5	62,5	177	54,5	
HBV	Y	333	3	37,5	86	29,5	0,62
	N		5	62,5	229	70,5	
Viral diseases (HCV/HBV)	Y	333	6	75	230	70,8	0,79
	N		2	25	95	29,2	
Transplanted Liver	whole	333	7	87,5	282	86,8	0,95
	partial		1	12,5	43	13,2	
Arterial Reconstructions	Y	333	2	25	52	16	0,49
	N		6	75	273	84	

Among the 8 patients with an aneurysm after the first LT, 4 (50%) were asymptomatic, 3 out of 5 patients (60%) with SA aneurysms, and 1 out of 3 (33%) with involvement of the HA.

### Symptomatic patients

Two symptomatic splenic aneurysms presented with an abrupt onset of hemorrhagic shock 5 and 8 days after LT: one patient underwent an urgent splenectomy and he is still alive and in good conditions; the second one died. One of the two symptomatic HA psudoaneurysms presented with an episode of hemobilia 56 days after transplantation, while the second had a severe deterioration in biochemical markers of liver function on the 12th postoperative day.

Overall, the symptoms of visceral symptomatic aneurysms appeared after a mean time of 19±27 days after LT, whereas the median time was 9 days after LT; In conclusion, in our experience, the symptoms of such aneurysms appear very early after LT.

### Asymptomatic patients

The visceral aneurysms were discovered incidentally with CT scans performed for other reasons, after 18,3±2 months, with a median time of 2,9 months.

An incidental diagnosis was performed with CT scan in 4 cases. Intraoperative diagnosis was made in 2 patients with haemorrhagic shock. In 2 patients there was time to perform US scan, CT scan and arteriography and after that elective surgery could be performed.

With regard to the therapy, among 5 splenic aneurysms, 2 had no treatment (LT 813-839) – in one patient the SA had already been ligated at LT, in order to improve the HA flow; 3 underwent splenectomy, an unsuccessful laparotomy and a distal splenopancreatectomy with construction of an hepato-aortic conduit – 5, 8 and 21 days after LT respectively (LT 744, 315 e 806).

Among 3 patients with HA aneurysms, one of them (LT 543) – affected by an intrahepatic aneurysm and an aneurysm of a previous aorto-hepatic conduit – underwent a re-transplantation with total hepatectomy and partial resection of the conduit with a new arterial anastomosis. The patient died 2 years later for HCV recurrence. The others (LT 731 and 444) underwent a resection and an extra-anatomical HA reconstruction with an aorto-hepatic conduit. Both of them are still alive and in good conditions.

Symptomatic and asymptomatic SA complications appeared after a mean (average) time of 40±56 days after LT (median 32), while those of the HA after 23±37 months (median 23 months). However, sympomatic aneurysms appeared early: 5 and 8 days after LT for ruptured SA aneurysms, and 10 and 56 days after LT for HA pseudoaneurysms.

Overall, only one (12,5%) out of 8 patients after LT died of causes directly related to a ruptured visceral aneurysm.

## Discussion

This study considers separately two specific categories of patients:

1. liver transplant candidates, i.e. end-stage liver disease patients, a subclass of severe liver disease patients. Patients with liver disease but not suitable for LT were not evaluated in this study.
2. liver transplanted patients, rarely studied in non-transplant units.

The aim is not to compare those two groups but to add data concerning visceral aneurysms in these two distinct populations.

### 1) Pre-transplant patients

The incidence of visceral aneurysms in our patients with severe cirrhosis was quite small (2.6%), This rate is probably realistic, if we consider the autopsy data for the general population (0.2 to 0.8%) [9]–[10] and the different data reported in previous studies on patients with cirrhosis (7–50%) [1]–[2]. Probably in our study some aneurysms were missed- frequently in cirrhotic patients the SA is long and tortuous and it may mask some small aneurysms. - anyway rates over 10% seem too high.

SA aneurysms occurred only in cirrhotic patients (9 of 9, i.e. 100%). This is consistent with published observations [11].

The analysis of etiological factors provided interesting information despite the limited number of aneurysms, considering that most of the articles appeared in literature included no more than 20 cases each [12], [13].

The female gender was more likely to be affected by aneurysms, with an odds ratio of 2.9 at univariate analysis. Although the female predominance was not significant at multivariate analysis; this trend is consistently reported in literature [14]. The reason maybe linked to the structural components of the arteries and to the effect of estrogens during pregnancy or during the menstrual cycle on the elastic tunica of the vascular wall [15].

The longitudinal diameter of the spleen was a significant risk factor at the univariate analysis but not at the multivariate one. The figure appears to be related to the severity of portal hypertension and its hemodynamic changes [3–16–17–18–19].

The absence of statistically significant relations at univariate analysis with liver function or specific scores (transaminases, INR, MELD and A.P.R.I. Scores) suggests that there is no evident relationship between liver function, hemodynamic changes and aneurysms' onset.

The absence of a relation between HBV and HCV infections and the onset of aneurysms suggests that the hemodynamic changes induced by cirrhosis are independent of the etiology. The data reported by some authors [14]–[20] of an increased prevalence of HBV positive patients were probably biased by heterogeneity of patients undergoing LT.

Multivariate analysis of factors believed to be related to the onset of visceral aneurysms in patients with cirrhosis showed a statistical significance for pre-transplant values of amylase, a previous beta-blocker therapy and TACE.

Hyperamylasaemia could be related with mild pancreatitis which could cause vascular damage and weakening of the SA [21]–[22]. This finding has already been observed in other studies [13].

The beta-blocker therapy could also have some explanations: first of all the beta-blockers are given to patients with the worst portal hypertension; in addition the visceral vasodilatation induced by beta-blockers might not be enough to balance the increased visceral blood flow. The relationship with the Trans-arterial chemo-embolization (TACE) is less clear: these treatments affect the HA and its branches, so a relationship with the splenic aneurysm of our cirrhotic population is unclear. This may be due to vasospasm, induced by intra-arterial catheters, and increasing the SA wall stress with increased risk of aneurysm formation.

In conclusion, factors such as levels of amylase, previous beta-blocker therapy and TACE, need additional studies to confirm their importance.

As reported in literature [23], visceral aneurysms are generally asymptomatic. This observation was confirmed in our series. Notably, SA aneurysms of our cirrhotic patients were always asymptomatic.

According to the literature (24), angiography, that may be diagnostic as well as interventional, is the best way to diagnose a SA aneurysm (28). US scan has a low sensitivity while two-dimensional and three-dimensional CT scan and MRI scan have a better accuracy (6, 12, 26, 25). Aneurysms in our patients with cirrhosis were always detected with CT scan during the pre-transplant study. When CT scan revealed an aneurysm, this finding was confirmed by arteriography. In this study US scan, even with contrast (27), was more useful in HA abnormalities than in SA aneurysms.

The transplant factor influences the classic therapeutic approach of aneurysms in patients in waiting list for LT. Because of their small diameter (never more than 3 cm) and consequently the low risk of rupture, for fear of future adhesions or impairment of a vessel (the SA) which could be useful at the time of transplantation, the treatment of aneurysms is postponed until the moment of transplantation. Consequently, at that time, a splenectomy is needed [28]. If the splenectomy is difficult to be performed, as often happens, a proximal SA ligation is chosen; however, this procedure may be unsatisfactory: indeed, subsequent CT scans often show patency of these arteries not likely related to splenic vascular recanalization, but to retrograde flow from short gastric vessels; so a proximal and distal SA ligation should be preferred [29-9-3-4-5-9-35]

With regard to radiological procedures, in our hospital there is no such expertise to enable the use of arterial stents [30]–[31]. According to the literature, percutaneous endovascular-stent placement for visceral aneurysms seems a promising procedure but it is still anecdotal for HA and SA aneurysms (32, 33, 34); moreover, arterial embolization often provides only partial success and the problem of gastric recanalization remains.

### 2) Post-transplant patients

The rate of visceral aneurysms and pseudoaneurysms was 1.8%, which is similar, although marginally lower than other published series [6]. The aneurysms mainly affected the SA (5 out of 8, ie 62,5%), but 37,5% of cases involved the HA. This could stem from the arterial anastomosis of the transplant, and not from a purely hemodynamic disorder [6]–[11].

The analysis of risk factors registered only an increased prevalence in females (OR 3.5), a fact that can be explained with the same considerations previously reported concerning cirrhotic patients. No other statistically significant factors were found; the arterial reconstructions during the transplant, even if followed by increased risk of aneurysmal lesions (odds ratio 1.85), did not reach statistical significance.

Probably the variables considered for the study of transplanted recipients – including some information of the donor and graft – are inadequate to find a relation with the occurrence of aneurysms after transplantation. Certain surgical factors such as, for example, the type of liver used (whole or reduced) [12]–[35], and the execution of vascular reconstructions during transplantation, showed no statistically significant values as etiological factors in transplanted patients (Table 2). Some factors, as re-transplant, and biliary procedures, showed no significance, differently from other reported results [6–35–36–37]. HA pseudoaneurysms surely must have a difficult to identify technical explanation; for SA aneurysms factors such as peaks of arterial pressure or the use of amynes should be further investigated.

The clinical picture of transplanted patients was definitely different from that of cirrhotics. Among the 8 transplanted patients, four cases −60% of SA aneurysms and 33% of liver - were asymptomatic. An acute rupture occurred in 2 cases of SA aneurysms, that is in 25% of post-LT aneurysms, and in 40% of post-LT spleen aneurysms [6]–[21].

In contrast to the data from cirrhotic patients, in transplanted patients an incidental diagnosis occurred in only 50% of cases (4 patients).

Therapeutic procedures of post-LT aneurysms are complex. For SA aneurysms we have usually followed the classic pattern of laparotomy in case of emergency; the indication for radiological procedures in semi-elective or elective conditions should probably be increased. However, HA aneurysms always raise complex surgical problems that can only be handled by experienced surgeons: the construction of aorto-hepatic conduits is one of the most common treatment options [6]; in case of treatment failure, in contrast with splenic aneurysms, the consequence is an hepatic graft with no arterial flow, a condition leading to death or requiring re-transplantation.

### Conclusion

Visceral aneurysms represent a poorly studied clinical entity with only small series of observations in healthy subjects and in patients with liver disease. In particular, papers concerning visceral aneurysms in liver transplanted patients are very few. However, given the surge in transplant numbers in recent years −93,634 in Europe from 1968 to December 2009, i.e. about 6000 per year and just under a thousand per year in Italy alone - the disease relevance is growing, also because the clinical onset can be complex and dramatic.

Our analysis concerns patients with end-stage liver disease in waiting list for LT and patients transplanted over the last 10 years, therefore providing up-to date results.

Assuming that our findings are limited to cirrhotic patients in list for LT, and they cannot be extended to all categories of cirrhotics, the incidence in patients with cirrhosis was 2.6%; the female sex was more affected in both groups.

SA was involved in 100% of cases. The multivariate analysis of potential etiological factors indicates the importance of an increased amylase, a beta-blocker therapy and a previous TACE.

Splenic aneurysms in patients with cirrhosis were generally asymptomatic.

The treatment of visceral aneurysms in patients with liver disease waiting for a transplant does not always follow the classical protocol. This is because the treatment of choice for SA aneurysms, such as splenectomy, is a so-called “previous surgery” factor, and although it does not involve the hepatic hilum, it could represent a risk factor for bleeding during the transplant; and bleeding is a well known risk factor for survival after LT. On the contrary, splenectomy, when performed in cirrhotics with low platelet counts, could improve this condition, with enhanced hemostasis.

The limited practice of SA embolization in cirrhotic patients is caused by the possible use of this artery during the transplant to ensure the arterial supply to the transplanted liver. So the treatment of SA aneurysms – the only one detected in our series of cirrhotics – consisted in standby and a ligation or a splenectomy during the LT. In only a few cases, because of the complexity of the transplantation involved, such treatments were not performed.

In liver transplanted patients the incidence of visceral aneurysms and pseudoaneurysms was 1,8% and the HA was affected in 25% of cases. In addition to the predisposition of the female sex, there were no other statistically significant factors related to the onset of visceral aneurysms; the arterial reconstructions during the transplant, even if followed by increased risk of aneurysmal lesions (odds ratio 1.85), did not reach statistical significance. Ruptures occurred in 25% of cases. Aneurysms of the HA presented with hemobilia or liver enzyme abnormalities, and often required complex surgical reconstruction. Severe complications occurred within 2 weeks from LT, treated with urgent splenectomy. Not urgent patients were treated with semi-elective ligations of the SA, whereas HA aneurysms can be risky and need more complex arterial reconstructions, whose failure may lead to a retransplant.

A specific issue has been the persistent patency of SA on CT scan after proximal ligation that can be due to spleno-gastric arteries. An option would be a splenectomy, or a proximal and distal ligation of the SA, or the section of gastro-splenic arteries associated with proximal ligation of the SA.

A spleen-sparing treatment of splenic aneurysms should always be considered as splenectomy exposes to infection risks, particularly in immunocompromised patients, even though published data on the subject are scarse. On the other hand, splenectomy may allow an early start of antiviral therapies.

Our retrospective study may have the weakness intrinsic in this type of studies: incomplete data collection. Considering a 10-year period has certainly reduced the problem, but the limits of this approach are still present.

This study explores some features of LT rarely considered in reviews.
